# Supplementary material for: Integrating Casein Complex SNPs Additive, Dominance and Epistatic Effects on Genetic Parameters and Breeding Values Estimation for Murciano-Granadina Goat Milk Yield and Components
Source: Genes (Basel). 2020 Mar 14;11(3):309. doi: 10.3390/genes11030309 (PMC7140789; doi:10.3390/genes11030309)
Supplement: Supplementary file 1 [file genes-11-00309-s001.zip › Supplementary Table S2.docx]

**Supplementary Table S2.** Summary of the results of epistatic relationship through Nonlinear Canonical Correlation Analysis, SNPs explaining intergroup variability and reinforcing epistatic interaction (Multiple fit>0.1), alleles for each SNP, their relative frequency and dominance ratios.

| **Gene** | **NLCC Dimension** | **SNPs explaining intergroup variability and reinforcing epistatic interaction (Multiple fit>0.1)** | **Alelles for each SNP** | **Relative frequency and dominance ratios** |
| --- | --- | --- | --- | --- |
| *CSN1S2* | 1 | from SNP1 to 6, 8, 10, 14 and 15 | A/C, T/C, G/C, G/C, G/A, C/T, A/G, A/G, G/A, G/T, T/C, respectively. | When the relative frequencies for these alleles are sorted from highest to lowest, we get the following series C, T, G and A. When analyzing the dominance ratios (using the > sign representing the dominance effects of one allele on the next), we found that A>C and G, for SNP1 presented a significant relationship (P<0.01) with milk yield, while SNP8 presented a significant relationship (P<0.01) with somatic cell count, respectively. G> A and T, for SNP4 and SNP14 presented a significant relationship (P<0.01) with lactose content, respectively. C> T, for SNP5 presented a significant relationship (P<0.01) with milk yield and lactose content, while T>C for SNP15 presented a significant relationship (P<0.01) with lactose content. |
| *CSN1S2* | 2 | SNP2, 3, 8, 10, 13 to 15, 17 and 18 | C/T, G/C, A/G, G/A, C/T, G/T, T/C, G/C and C/T, respectively | When analyzing the dominance ratios (With the sign > representing the dominance effects of one allele on the next), we found C>T, for SNP2 and SNP18, a significant effect (P<0.01) was found on the percentage of protein and lactose, respectively, while G> C, for SNP3 and SNP17 reported a significant association with protein percentage and milk yield, respectively. For *CSN1S2* SNP8, A allele presented a significant dominant character over G, for protein and somatic cells count (P<0.01). SNP10 reported a significant dominance effect of allele G over A for milk yield and all components (fat, protein, dry matter, lactose percentage) and somatic cells count. SNP13 reported a significant dominance relationship of C over T for protein and lactose content (%) while SNP15 reported the same significant dominance allelic behavior but only on lactose component. SNP14 reported a significant dominance effect of G allele over T for lactose. |
| *CSN1S1* | 1 | SNP19, 20, 22, 24 to 26, 28 and 31 | A/G, G/A, T/C, A/G, A/G, A/G, G/C and T/C, respectively | When the relative frequencies for these alleles were sorted from highest to lowest, the following series was obtained A=G and C=T (with the equal sign meaning same frequency). When analyzing the dominance relationships (With the sign > representing the dominance effects of one allele on the next) we found that no dominance effect was reported for SNP19, SNP22 and SNP28. For SNP20 G presented a significant dominant relationship over A (P<0.01) on protein content and milk yield. The same situation was described for SNP24 and somatic cell count, and SNP26 and lactose content, respectively. On the contrary, A>G, for SNP25 presented a significant relationship (P<0.01) with protein content, while alleles T>C for SNP31 presented a significant effect (P<0.01) on protein content. |
| *CSN1S1* | 2 | SNP19, 20, 22, 24, 25, 28, 29 and 31 | G/A, G/A, T/C, A/G, A/G, G/C, A/G and T/C, respectively | On the one hand, the relative frequencies for both alleles were the same in all cases and the same circumstances described above were replicated, with the exception that no repercussion of SNP26 was reported. On the other hand, despite SNP29 had repercussion on the epistatic interaction (it did not in dimension 1 for *CSN1S1*), its alleles did not report any dominance effect. |
| *CSN2* | 1 | SNP36 | C over T | For this dimension 1, the allelic combination CT resulted in the highest levels for somatic cell count (with C being dominant over T). |
| *CSN2* | 2 | SNP35 | G = A | No dominance relationship was reported for the alleles involved. |
| *CSN3* | 1 | SNP39, 41, 42, 46 and 47 | C/T, A/T, .-/AATC, A/T and G/C | When the relative frequencies for these alleles are sorted from highest to lowest T = C y AATC = .-. When analyzing the dominance relationship between alleles (With the sign > representing the dominance effects of one allele on the next) we have that T>C, for SNP39 presented a significant relationship (P<0.01) with lactose content. Simultaneously, there was a dominance relationship of T> A, for SNP41 presenting a significant relationship with lactose content. A allele reported a significant dominance effect over T for SNP46 on fat content and for SNP47, G was dominant over C for lactose content. |
| *CSN3* | 2 | SNPs46 and 47 | A/T and G/C, respectively | When analyzing the dominance relationships (With the sign > representing the dominance effects of one allele on the next) A>T alleles for the SNP46 reported a significant relationship with fat content. On the contrary, we found a significant relationship for the G>C allele from SNP47 with lactose content. |
